# Supplementary material for: A fault-intrusion-tolerant system and deadline-aware algorithm for scheduling scientific workflow in the cloud
Source: PeerJ Comput Sci. 2021 Nov 2;7:e747. doi: 10.7717/peerj-cs.747 (PMC8576554; doi:10.7717/peerj-cs.747)
Supplement: Supplemental Information 6 [file peerj-cs-07-747-s006.zip › Myproject1.1/docs/org/cloudbus/cloudsim/examples/power/random/RandomRunner.html]

RandomRunner


JavaScript is disabled on your browser.


- Overview
- Package
- Class
- Use
- Tree
- Deprecated
- Index
- Help

- Prev Class
- Next Class

- Frames
- No Frames

- All Classes

- Summary:
- Nested |
- Field |
- Constr |
- Method

- Detail:
- Field |
- Constr |
- Method


org.cloudbus.cloudsim.examples.power.random

## Class RandomRunner

- java.lang.Object
- - org.cloudbus.cloudsim.examples.power.RunnerAbstract
  - - org.cloudbus.cloudsim.examples.power.random.RandomRunner

- ---

    

  ```
  public class RandomRunner
  extends RunnerAbstract
  ```

  The example runner for the random workload.
  If you are using any algorithms, policies or workload included in the power package please cite
  the following paper:
  Anton Beloglazov, and Rajkumar Buyya, "Optimal Online Deterministic Algorithms and Adaptive
  Heuristics for Energy and Performance Efficient Dynamic Consolidation of Virtual Machines in
  Cloud Data Centers", Concurrency and Computation: Practice and Experience (CCPE), Volume 24,
  Issue 13, Pages: 1397-1420, John Wiley & Sons, Ltd, New York, USA, 2012

  Since:
  :   Jan 5, 2012

- - ### Field Summary

    - ### Fields inherited from class org.cloudbus.cloudsim.examples.power.RunnerAbstract

      `broker, cloudletList, hostList, vmList`
  - ### Constructor Summary

    Constructors

    Constructor and Description

    `RandomRunner(boolean enableOutput,
    boolean outputToFile,
    java.lang.String inputFolder,
    java.lang.String outputFolder,
    java.lang.String workload,
    java.lang.String vmAllocationPolicy,
    java.lang.String vmSelectionPolicy,
    java.lang.String parameter)`
  - ### Method Summary

    Methods

    | Modifier and Type | Method and Description |
    | --- | --- |
    | `protected void` | `init(java.lang.String inputFolder)` Inits the simulation. |

    - ### Methods inherited from class org.cloudbus.cloudsim.examples.power.RunnerAbstract

      `getExperimentName, getVmAllocationPolicy, getVmSelectionPolicy, initLogOutput, isEnableOutput, setEnableOutput, start`
    - ### Methods inherited from class java.lang.Object

      `clone, equals, finalize, getClass, hashCode, notify, notifyAll, toString, wait, wait, wait`

- - ### Constructor Detail


    - #### RandomRunner

      ```
      public RandomRunner(boolean enableOutput,
                  boolean outputToFile,
                  java.lang.String inputFolder,
                  java.lang.String outputFolder,
                  java.lang.String workload,
                  java.lang.String vmAllocationPolicy,
                  java.lang.String vmSelectionPolicy,
                  java.lang.String parameter)
      ```

      Parameters:
      :   `enableOutput` -
      :   `outputToFile` -
      :   `inputFolder` -
      :   `outputFolder` -
      :   `workload` -
      :   `vmAllocationPolicy` -
      :   `vmSelectionPolicy` -
      :   `parameter` -
  - ### Method Detail


    - #### init

      ```
      protected void init(java.lang.String inputFolder)
      ```

      **Description copied from class: `RunnerAbstract`**

      Inits the simulation.

      **Specified by:**
      :   `init` in class `RunnerAbstract`

      Parameters:
      :   `inputFolder` - the input folder


- Overview
- Package
- Class
- Use
- Tree
- Deprecated
- Index
- Help

- Prev Class
- Next Class

- Frames
- No Frames

- All Classes

- Summary:
- Nested |
- Field |
- Constr |
- Method

- Detail:
- Field |
- Constr |
- Method
